# Prevention of CD8 T Cell Deletion during Chronic Viral Infection

**DOI:** 10.3390/v13071189

**Published:** 2021-06-22

**Authors:** David G. Brooks, Antoinette Tishon, Michael B. A. Oldstone, Dorian B. McGavern

**Affiliations:** 1 Viral Immunobiology Laboratory, Department of Immunology and Microbial Science, The Scripps Research Institute, 10550 North Torrey Pines Rd., La Jolla, CA 92037, USA; atishon@scripps.edu (A.T.); mbaobo@scripps.edu (M.B.A.O.); 2 Princess Margaret Cancer Center, University Health Network, Toronto, ON M5G 2M9, Canada; 3 Department of Immunology, University of Toronto, Toronto, ON M5S 1A8, Canada; 4 Viral Immunology and Intravital Imaging Section, National Institute of Neurological Disorders and Stroke, The National Institutes of Health, 10 Center Drive, Bethesda, MD 20895, USA

**Keywords:** LCMV, chronic infection, CD8 T cells, CD4 T cell help, T cell deletion, exhaustion, costimulation, antiviral therapy, ribavirin

## Abstract

During chronic viral infections, CD8 T cells rapidly lose antiviral and immune-stimulatory functions in a sustained program termed exhaustion. In addition to this loss of function, CD8 T cells with the highest affinity for viral antigen can be physically deleted. Consequently, treatments designed to restore function to exhausted cells and control chronic viral replication are limited from the onset by the decreased breadth of the antiviral T cell response. Yet, it remains unclear why certain populations of CD8 T cells are deleted while others are preserved in an exhausted state. We report that CD8 T cell deletion during chronic viral infection can be prevented by therapeutically lowering viral replication early after infection. The initial resistance to deletion enabled long-term maintenance of antiviral cytolytic activity of the otherwise deleted high-affinity CD8 T cells. In combination with decreased virus titers, CD4 T cell help and prolonged interactions with costimulatory molecules B7-1/B7-2 were required to prevent CD8 T cell deletion. Thus, therapeutic strategies to decrease early virus replication could enhance virus-specific CD8 T cell diversity and function during chronic infection.

## 1. Introduction

At the beginning of what will become a chronic infection, heightened and sustained antigenic signaling and inflammatory factors enhance the expression of immunosuppressive cytokines and multiple inhibitory receptors/ligands (e.g., IL10, PD1, PDL1) that program and maintain T cell exhaustion and viral persistence [1,2,3,4]. The exhaustion of antiviral T cell function is observed during many chronic viral infections, including HIV and hepatitis B and C virus (HBV, HCV) infections of humans, lymphocytic choriomeningitis virus (LCMV) infection of rodents, and in response to many cancers [3], suggesting that shared mechanisms triggered by increased and/or sustained antigen regulate T cell inactivation. Although antiviral T cells with diminished function can remain in the immune repertoire, CD8 T cells with the highest affinity for viral antigens are sometimes physically deleted and thus absent throughout the infection [5,6,7]. Since virus-specific T cells continue to maintain some effector function and exert long-term pressure on virus replication, they often serve as targets for immune restorative therapies [3]. Unfortunately, the deletion of high affinity CD8 T cells can greatly hamper such therapeutic efforts and impede the host in its fight against a chronic virus. Understanding the mechanisms governing T cell exhaustion and deletion is important if we intend to eradicate persistent viral infections. We have already begun to identify distinct mechanisms that control of the functional states of antiviral T cells. For example, we demonstrated previously that loss of cytokine production and effector functions can be separated from the process of cellular expansion [8]. It therefore stands to reason the physical deletion of T cells might also be preventable despite functional exhaustion.

Deletion of high-affinity CD8 T cells has multiple potential consequences. First, it results in depletion of effector cells well equipped to fight infection, thereby limiting the breadth, magnitude, and efficacy of the antiviral response. Second, with high-affinity antiviral CD8 T cells removed, therapeutic interventions designed to control established chronic infections by resurrecting T cell responses are limited to restoring function to T cells of a low to moderate affinity. At the very least, preservation of otherwise deleted T cells has the potential to broaden the long-term antiviral response by providing more cells. Thus, methods that prevent the deletion of high-affinity CD8 T cells could be of great benefit to both the endogenous and therapeutic control of chronic viral infections.

The mechanisms that regulate deletion during viral infection are still unclear, although levels of antigen stimulation at the epitope level is higher for cells that are deleted [9], leading to the theory that cognate peptide-MHC density on the cell surface determines physical deletion versus maintenance of functionally exhausted T cells. Virus-specific CD4 T cells also participate in the fate of CD8 T cells. For example, CD4 T cell help is required to sustain virus-specific CD8 T cells during chronic infection [10,11], and re-establishment of CD4 T cell responses [12], specifically of CD4 Th1 cells [13], in the midst of an established chronic infection can prevent the progressive numerical and functional decay of virus-specific CD8 T cells. However, the early role of CD4 T cells in the priming of CD8 T cells, during what will eventually become a chronic infection, is less clear. In fact, early functional rescue of CD4 T cells during persistent viral infection by the administration of antiviral therapy still leads to CD8 T cell exhaustion [8].

In this study, we sought insights into the mechanisms that give rise CD8 T cell deletion during a persistent viral infection. We demonstrate that distinct from CD8 T cell functional exhaustion, the physical deletion of high-affinity LCMV-NP_396–404_ specific CD8 T cells in chronic LCMV infection is due to increased virus infection and the loss of costimulatory signals. Therapeutic reduction of viral replication with the antiviral drug Ribavirin (Rb) prevented deletion of high affinity virus-specific CD8 T cells, which were then maintained in a functional state long after Rb treatment was ceased. Importantly, deletion of CD8 T cells was not linked solely to levels of virus replication. Instead, continued CD4 T cell help and prolonged costimulatory interactions with B7-1 (CD80) and B7-2 (CD86) were also needed to prevent deletion.

## 2. Materials and Methods

### 2.1. Mice and Virus

C57BL/6 (H-2^b^, Thy1.2+) mice were from The Rodent Breeding Colony at the Scripps Research Institute. CD4−/− mice were bred from homozygous breeding pairs obtained from The Jackson Laboratory (Bar Harbor, ME, USA). The LCMV-GP_61–80_-specific CD4+ T cell receptor (TCR) transgenic (SMARTA) mice have been described previously [14] and were gifts from Hans Hengartner and Rolf Zinkernagel (University Hospital of Zurich, Zurich, Switzerland). All mice were housed under specific pathogen-free conditions. Mouse handling conformed to the requirements of The Scripps Research Institute Animal Research Committee. Mice were infected intravenously with 2 × 10^6^ plaque forming units (PFU) of LCMV-Arm or LCMV-Cl13. Virus stocks were prepared as described [14]. Viral titers were determined by plaque formation on Vero cells [14].

### 2.2. Flow Cytometry

Total splenocytes were stimulated for 5 h with 2 µg/mL of the MHC class I restricted LCMV-NP_396–404_ peptide (>99% pure; Synpep, Dublin, CA, USA) in the presence of 50 U/mL recombinant murine IL-2 (R&D Systems, Minneapolis, MN, USA) and 1 mg/mL brefeldin A (Sigma, St. Louis, MO, USA). The absolute number of NP_396–404_-specific CD8 T cells was determined by multiplying the frequency of IFNγ+ cells by the total number of splenocytes.

### 2.3. Ribavirin Treatment

Mice were treated with 100 mg/kg Rb (1-b-D-ribofuranosyl-1,2,4-triazole-3-carboxamide, Virazole; ICN Pharmaceuticals, Costa Mesa, CA, USA) in PBS. We observed no toxicity with the use of Rb. Treatment was administered once daily intraperitoneally (i.p.) on days 1–8 after LCMV infection. Control mice were injected in parallel with PBS (vehicle-only control).

### 2.4. Costimulation Blockade

Mice received 250 µg CTLA4-Ig (Sigma, non-cytolytic) i.p. once daily or every other day on days 4–8 after infection. Control mice were treated in parallel with 250 µg matched mouse IgG2a isotype control antibody (BD Pharmingen, La Jolla, CA, USA).

### 2.5. In Vitro and In Vivo Cytotoxicity Assays

For in vitro ^51^Cr release assays, splenocytes on day 9 after the indicated infection were incubated for 5 h with either unlabeled MC57 cells, LCMV-GP_33–41_ peptide labelled MC57 cells, or NP_396–404_ peptide labeled MC57 cells. For in vivo CTL assays, splenocytes from naïve C57BL/6 mice were either left unlabeled or were labeled with 2 µg/mL of the LCMV-NP_396–404_ peptide. These target cell populations were then differentially labeled with CFSE (2.5 or 0.25 µg/mL) and transferred i.v. (5 × 10^6^ cells of each population) into mice previously infected with LCMV. Fifteen hours post transfer, splenocytes were analyzed for CFSE expression by flow cytometry. Percent lysis = [1 − (ratio NP396 to unlabeled)] × 100.

### 2.6. Statistical Analysis

Statistical analyses were performed using GraphPad Prism 6 software (GraphPad Software, Inc., San Diego, CA, USA). In the line and bar graphs the error bars indicate standard deviation.

## 3. Results

### 3.1. Lowering Virus Titers during Early Chronic Infection Preserves High-Affinity LCMV-NP_396–404_ Dpecific CD8 T Cells

**Figure 1:**
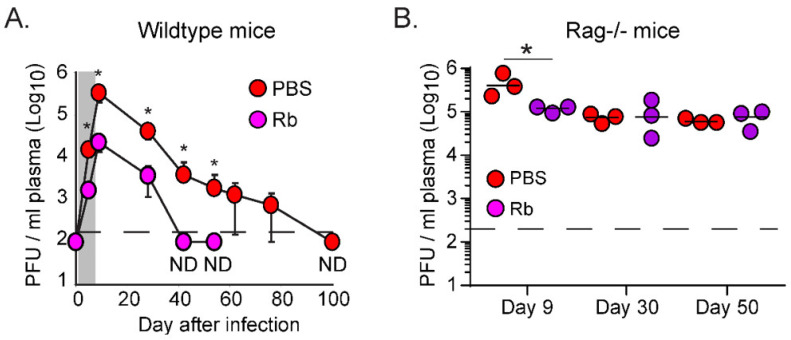
Accelerated control of chronic viremia following Rb therapy is dependent on adaptive immune responses. (**A**) Plasma viral titers from LCMV-Clone 13 (Cl13) infected C57BL/6 mice either PBS contro-treated (red circles) or treated with Ribavirin (Rb; purple circles) were determined at the indicated time point. Data are expressed as PFU per milliliter of plasma. Shaded area indicates time of Rb treatment (day 1–8 after infection). Each time point represents the average ± standard deviation (SD) of 4 mice per group. ND = not detected. (**B**) Infectious virus in the plasma of LCMV-Cl13 infected Rag−/− mice either PBS-treated or treated with Rb. Each circle represents a single animal. Data are representative of 2 experiments with 3–4 mice per group. * *p* < 0.05; one-way ANOVA.

To determine how early changes in virus replication affect T cell responses, we infected mice with LCMV-Clone13 (Cl13) to generate a chronic infection [15]. Mice were then treated daily with the antiviral drug Ribavirin (Rb) beginning one day after infection until day 8 [8]. As we demonstrated previously [8], early antiviral therapy with Rb rapidly lowered virus titers, leading to enhanced control of virus replication after the treatment was discontinued (Figure 1A). To assess whether the accelerated viral control required adaptive immunity or resulted solely in the initial lowering of viral titer, we infected Rag−/− mice (lacking B and T cells) with LCMV-Cl13. Even in the absence of B cells and T cells, Rb treatment led to a 4-fold decrease in viral replication (Figure 1B). However, virus titers remained similarly elevated in PBS vs. Rb treated Rag−/− mice following withdrawal of therapy (Figure 1B), indicating that the accelerated viral clearance conferred by Rb treatment in wild type (WT) mice was reliant on adaptive immune responses.

**Figure 2:**
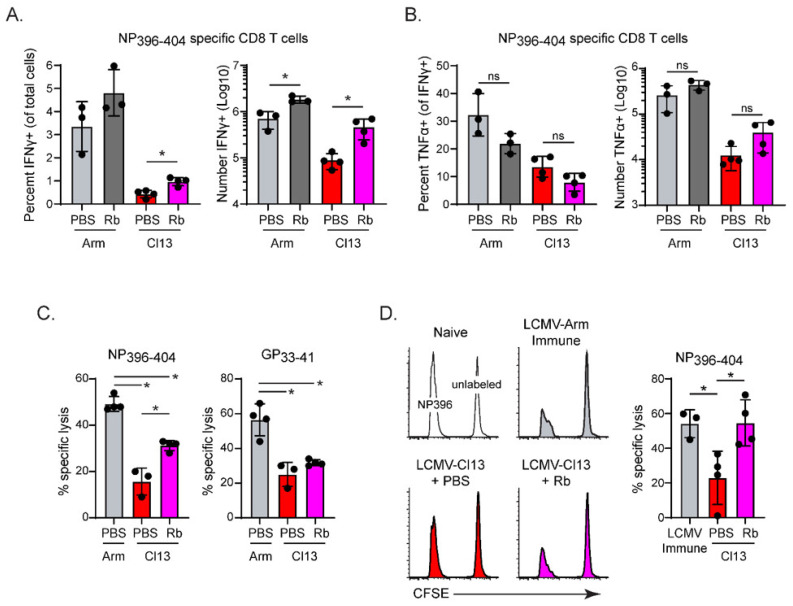
Early prevention and long-term functional rescue of high-affinity CD8 T cells. (**A**,**B**). Splenocytes from PBS-treated or Rb-treated LCMV-Arm or LCMV-Cl13 infected C57BL/6 mice were analyzed on day 9 after infection. Graphs illustrate (**A**) the frequency and number of IFNγ-producing, and (**B**) of TNFα and IFNγ double producing NP_396–404_-specific CD8 T cells following ex vivo peptide restimulation. The bars represent the average ± SD of 4 mice (individual circels) in each group and represent multiple experiments. *, *p* < 0.05. (**C**) In vitro ^51^Cr release activity against target cells coated with LCMV-NP_396–404_ peptide or LCMV-GP_33–41_ peptide (effector:target ratio of 50:1) on day 9 post infection. (**D**) In vivo CTL assay (day 50 after infection). Peptide-unlabeled or NP_396–404_ peptide-labeled target cells (splenocytes) were co-transferred into LCMV-naïve mice, LCMV-Arm immune mice (day 50 after infection) or LCMV-Cl13 infected mice (day 50 after infection) that were either PBS-treated or had been treated with Rb on days 1 to 8 after infection. Histograms show a single mouse with each condition, and the bar graph indicates the percent NP_396–404_-specific lysis. The bar graph shows the average ± SD of 3 (Arm infection) or 4 (Cl 13 and Cl 13 + Rb infections) mice per group. The circles on each bar represent individual mice. * *p* < 0.05; student’s t-test (unpaired, two-tailed).

We next sought to determine how the initial decrease in virus titers altered the virus-specific CD8 T cell response. We have shown previously that LCMV-specific CD4 T cells robustly resisted exhaustion when virus titers were decreased by early Rb treatment, with a smaller functional restoration of LCMV-GP_33–41_-specific CD8 T cells [8]. Interestingly, there was an increase in both the frequency and number of the IFNγ-producing high-affinity LCMV-NP_396–404_-specific CD8 T cells following Rb treatment (Figure 2A). Across experiments, the number of IFNγ-producing LCMV-NP_396–404_-specific CD8 T cells was increased approximately 2–5-fold in Rb treated compared to untreated LCMV-Cl13 infected mice (Figure 2A). Rb treatment also increased the number of IFNγ-producing LCMV-NP_396–404_-specific CD8 T cells following acute LCMV-Arm infection, albeit to a lesser extent than during chronic LCMV-Cl13 infection (Figure 2A). Despite the increased number of IFNγ-producing LCMV-NP_396–404_ specific CD8 T cells in Rb treated chronic LCMV-Cl13 infected mice, the frequency of IFNγ/TNFα co-producing cells was not increased (Figure 2B), suggesting that preservation of the response was still accompanied by loss of polyfunctional cytokine potential. However, preservation of LCMV-NP_396–404_ specific CD8 T cells following early Rb treatment resulted in increased NP_396–404_-specific lysis (CTL) at day 9 post-infection (likely reflecting the increased number of NP396–404-specific cells), whereas this treatment had a minimal effect on the LCMV-GP_33–41_ specific CTL activity (Figure 2C). These data indicate that Rb physical rescues high-affinity NP_396_-specific CD8 T cells and preserves their CTL activity.

We next assessed whether the early preservation of LCMV-NP_396–404_-specific CD8 T cells resulted in long-term CTL lytic activity by performing an in vivo CTL assay. Mice were treated with vehicle or Rb on days 1–8 after LCMV-Cl13 infection. In parallel, mice were left naïve to LCMV infection or infected with acutely resolved LCMV-Arm to generate LCMV-immune mice. Fifty days after infection, unlabeled or NP_396–404_-peptide labeled splenocytes from naïve mice were transferred into the LCMV-naïve mice, LCMV-Arm ‘immune’ and LCMV-Cl13 infected mice. LCMV-Cl13 infected mice had diminished NP396–404 directed CTL lysis compared to LCMV-immune mice, although residual lysis was still observed in comparison to LCMV-naïve mice (Figure 2D). Importantly, LCMV-Cl13 infected mice initially treated with Rb exhibited sustained CTL lytic activity against the LCMV NP_396–404_ epitope that was similar to the level observed in LCMV-Arm immune mice (Figure 2D), indicating that preventing the initial deletion of the NP396–404 CD8 T cell response resulted in preserved lytic activity of these cells.

### 3.2. CD4 T Cells Are Required to Preserve High-Affinity LCMV-NP_396–404_ Specific CD8 T Cells

**Figure 3:**
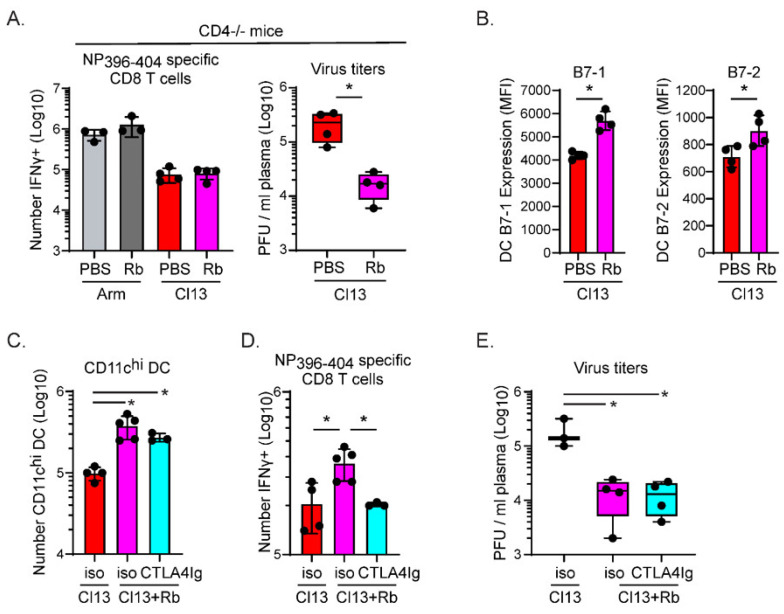
CD4 T cells and prolonged costimulation are required in combination with lower virus replication to prevent CD8 T cell deletion. (**A**). Splenocytes from PBS-treated or Rb-treated LCMV-Arm or LCMV-Cl13 infected CD4−/− mice were analyzed on day 9 after infection. The bar graph represents the number of IFNγ-producing NP_396–404_ specific CD8 T cells and is the average ± SD of 4 mice per group. Box and whisker indicate day 9 virus titers in PBS-treated and Rb-treated CD4−/− mice infected with LCMV-Cl13. In the box and whisker plots, the midline represents the median and the error bars the minimum and maximum values (whiskers). Circles indicate individual mice. (**B**). Bars indicate the mean fluorescence intensity (MFI) of B7-1 and B7-2 expression on splenic CD11c^hi^ dendritic cells in PBS treated or Rb-treated mice on day 9 after LCMV-Cl13 infection. Circles indicate the MFI in individual mice. (**C**–**E**). Splenocytes from PBS-treated or Rb-treated LCMV-Cl13 infected mice were analyzed on day 9 after infection. Mice were treated with PBS or Rb and then on days 4–8 after infection given CTLA4-Ig or an isotype antibody. Bars indicate (**C**) the number of CD11c^hi^ dendritic cells, **(D)** the number of IFNγ-producing NP396-specific CD8 T cells, and (**E**) plasma virus titers. Data are representative of 2 or more experiments with 3–5 mice per group. * *p* < 0.05; student’s t-test (unpaired, two-tailed).

A main effect of early antiviral Rb treatment and diminished viral replication following LCMV-Cl13 infection was the rescue of CD4 T cell function [8]. To determine the role of CD4 T cells in preserving LCMV-NP_396–404_-specific CD8 T cells, we used CD4-deficient mice. Consistent with a minimal impact of Rb treatment in enhancing NP_396–404_-specific CD8 T cells following acute LCMV-Arm infection, no alteration in the number of IFNγ-producing NP_396–404_-specific CD8 T cells was observed in Rb treated LCMV-Arm infected CD4−/− mice (Figure 3A). In contrast, the number of IFNγ-producing NP_396–404_-specific CD8 T cells were similarly deleted in Rb-treated and PBS-treated CD4−/− mice following LCMV-Cl13 infection (Figure 3A). Further, deletion occurred in Rb treated CD4−/− mice despite a substantial reduction in virus titers (Figure 3A). These data suggest that CD4 T cells are required to prevent deletion of IFNγ-producing high-affinity NP_396–404_-specific CD8 T cells in Rb treated mice secondary to the reduction in viral load.

### 3.3. Prolonged Costimulatory Interactions Are Required to Sustain Virus-Specific CD4 T Cells and High-Affinity LCMV-NP_396–404_ Specific CD8 T Cells

Decreased LCMV-Cl13 titers following Rb treatment coincided with upregulation of the costimulatory molecules B7-1 and B7-2 on dendritic cells (DCs; Figure 3B) and other antigen presenting cell (APC) populations (B cells and macrophages) [8], suggesting that prolonged or repeated costimulatory interactions during chronic viral infection might sustain T cells when virus loads are reduced. To evaluate the effect of prolonged/repeated costimulation, mice were infected with LCMV-Cl13, treated with the antiviral compound Rb to diminish viral replication, and then treated with CTLA4-Ig to specifically block CD28/CTLA4:B7-1/B7-2 interactions. CTLA4-Ig or mouse IgG2a isotype control antibody was administered beginning on day 4 after infection to avoid inhibiting the initial priming interactions [14]. The CTLA4-Ig used did not deplete cells, as evidenced by the similar numbers of DCs in Rb-treated LCMV-Cl13 infected mice given isotype or CTLA4-Ig, that were both higher than LCMV-Cl13 infection alone (Figure 3C). The preservation of LCMV-NP_396–404_-specific CD8 T cells afforded by early Rb treatment was now inhibited by blocking costimulation with CTLA4-Ig (Figure 3D). Importantly, the Rb+CTLA4-Ig treatment did not prevent the reduction in virus titers induced by Rb treatment alone (Figure 3E). These data suggest the decrease in virus titers alone was not sufficient to rescue NP396-specific CD8 T cells, but that prolonged costimulation was required after suppression of virus replication.

## 4. Discussion

Our studies demonstrate that deletion of high-affinity CD8 T cells during chronic viral infection can be prevented by decreasing early virus titers and enabling costimulatory B7.1/B7.2 interactions. Unlike functional inactivation, after which cells are physically present, deleted cells are absent throughout infection and cannot be resurrected. Consequently, treatments designed to restore function to inactivated cells [1] may have limited efficacy due to the extinction of high-affinity cells and the decreased breadth of the T cell response. Preventing deletion of high-affinity CD8 T cells creates the opportunity to restore and stimulate a broader repertoire of antiviral T cells to a control persistent viral replication. A major thrust of current therapeutic vaccination strategies is to restore function to inactivated T cells with the hope that they will control the chronic viral infection. It is important to note that these data utilized NP_396–404_ specific IFNγ production and not MCH I tetramer to quantify the NP_396–404_-specific CD8 T cells. Thus, although the deletion of NP_396–404_ reactive cells is prevented, it still remains to be determined whether this is due to survival of the cells and/or increased functionality. Importantly, our data indicate that if the early deletion of high-affinity CD8 T cells is abrogated, these cells can be functionally maintained and engaged throughout a state of viral persistence.

LCMV, like Lassa fever virus, is an Old-World arenavirus and is often used as a model of Lassa fever virus infection. In response to Lassa fever virus infection, survival versus death is associated with the amount of virus produced [16]. A recent study demonstrated that surviving individuals mounted a more robust Lassa virus-specific CD4 and CD8 T cell response relative to those that died from the disease [17,18]. Interestingly, Rb is effective in treating Lassa fever virus infection, and our data regarding the preservation of high affinity CTL in response to Rb provide another potential mechanism for how therapeutic control might be achieved in Lassa fever patients.

Functionally sustaining and enhancing CD4 T cell help during viral persistence (and particularly Th1 responses) maintains and enhances virus-specific CD8 T cells that are present throughout a chronic infection [12,13,19,20,21]. Nevertheless, virus-specific CD8 T cell exhaustion and deletion still occur at early time points post-infection despite the presence of CD4 T cells (albeit with attenuated function themselves) [10,11,14]. Importantly, our data demonstrate that CD4 T cells help prevent deletion of high-affinity NP396-specific CD8 T cells after virus titers are therapeutically decreased with Rb in the early stages of a chronic LCMV infection. Exactly how this early CD4 T cell help is delivered remains to be defined, but one possibility is increased IL-2 production by virus-specific CD4 T cells resulting from the diminished viral titers [8]. During HIV infection, deletion of high-affinity CD8 T cells inversely correlates with the benefit of early antiretroviral therapy and the maintenance of CD4 T cell activity [7]. Thus, the mechanisms leading to CD8 T cell deletion during states of heightened virus replication may be similar between rodent and human chronic infections.

Finally, our data suggest that mechanisms in addition to high levels of antigen exposure and prolonged/heightened TCR signaling result in T cell deletion [5,9]. Specifically, we observed that while deletion can be prevented by lowering virus titers with Rb, maintenance of these high-affinity CD8 T cells still requires additional interactions with costimulatory molecules. Thus, reduction of viral titers alone is insufficient when it comes to preventing deletion of high-affinity CD8 T cells. We identified sustained interactions with costimulatory molecules as one mechanism that helps prevent CTL deletion, but there are likely to be others. We postulate that decreasing viral replication with early antiviral therapy elevates costimulatory molecule expression on APCs, which provides CD8 T cells with the continued signals they need to survive despite viral persistence. The early preservation of these high-affinity CD8 T cells through decreased virus replication, augmented CD4 help, and enhanced costimulatory signals has important implications for virus control and immune restorative therapies during chronic viral infections.

## Data Availability

Reagent requests can be made to D.G.B.
